# Supplementary material for: Causal Relationship between Aging and Anorexia Nervosa: A White-Matter-Microstructure-Mediated Mendelian Randomization Analysis
Source: Biomedicines. 2024 Aug 16;12(8):1874. doi: 10.3390/biomedicines12081874 (PMC11351342; doi:10.3390/biomedicines12081874)
Supplement: Supplementary file 1 [file biomedicines-12-01874-s001.zip › Supplementary Figure/Figure S4/Singe SNP plot of MR analysis for TL to ubm-b-1874.pdf]

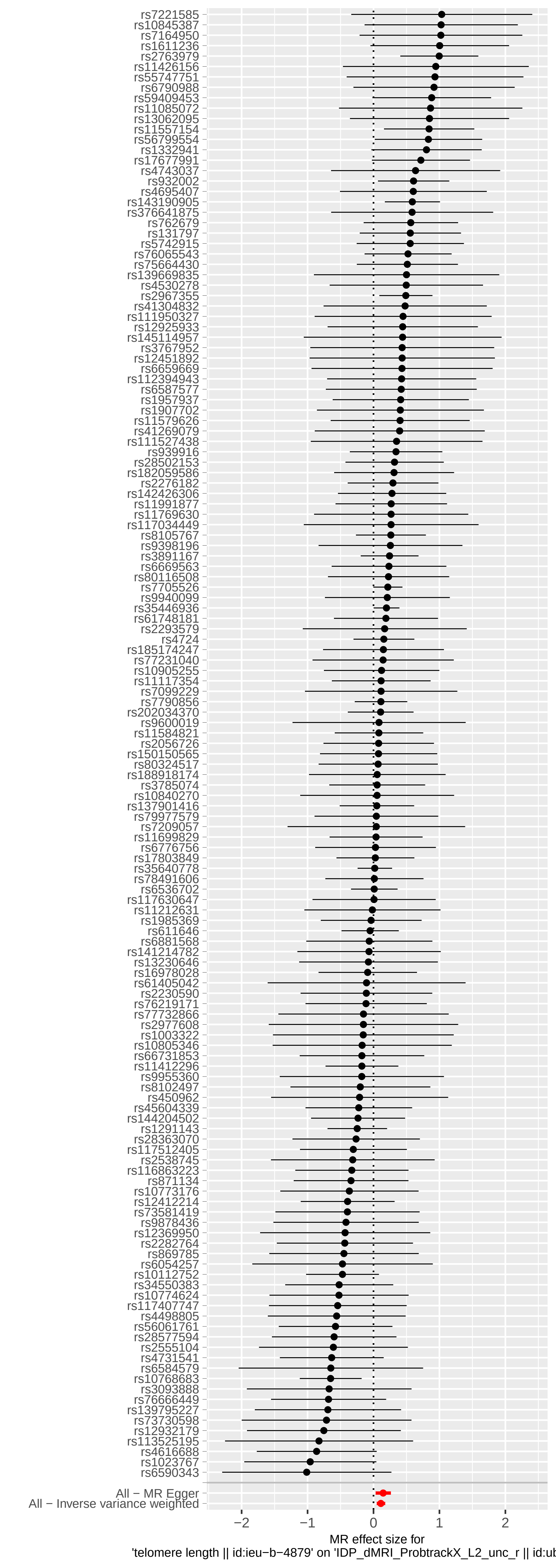
rs7221585
rs10845387
rs7164950
rs1611236
rs2763979
rs11426156
rs55747751
rs6790988
rs59409453
rs11085072
rs13062095
rs11557154
rs56799554
rs1332941
rs17677991
rs4743037
rs932002
rs4695407
rs143190905
rs376641875
rs762679
rs131797
rs5742915
rs76065543
rs75664430
rs139669835
rs4530278
rs2967355
rs41304832
rs111950327
rs12925933
rs145114957
rs3767952
rs12451892
rs6659669
rs112394943
rs6587577
rs1957937
rs1907702
rs11579626
rs41269079
rs111527438
rs939916
rs28502153
rs182059586
rs2276182
rs142426306
rs11991877
rs11769630
rs117034449
rs8105767
rs9398196
rs3891167
rs6669563
rs80116508
rs7705526
rs9940099
rs35446936
rs61748181
rs2293579
rs4724
rs185174247
rs77231040
rs10905255
rs11117354
rs7099229
rs7790856
rs202034370
rs9600019
rs11584821
rs2056726
rs150150565
rs80324517
rs188918174
rs3785074
rs10840270
rs137901416
rs79977579
rs7209057
rs11699829
rs6776756
rs17803849
rs35640778
rs78491606
rs6536702
rs117630647
rs11212631
rs1985369
rs611646
rs6881568
rs141214782
rs13230646
rs16978028
rs61405042
rs2230590
rs76219171
rs77732866
rs2977608
rs1003322
rs10805346
rs66731853
rs11412296
rs9955360
rs8102497
rs450962
rs45604339
rs144204502
rs1291143
rs28363070
rs117512405
rs2538745
rs116863223
rs871134
rs10773176
rs12412214
rs73581419
rs9878436
rs12369950
rs2282764
rs869785
rs6054257
rs10112752
rs34550383
rs10774624
rs117407747
rs4498805
rs56061761
rs28577594
rs2555104
rs4731541
rs6584579
rs10768683
rs3093888
rs76666449
rs139795227
rs73730598
rs12932179
rs113525195
rs4616688
rs1023767
rs6590343
All − MR Egger
All − Inverse variance weighted
−2
−1
0
1
2
MR effect size for
'telomere length || id:ieu−b−4879' on 'IDP_dMRI_ProbtrackX_L2_unc_r || id:ub
